# Degradation Type-Aware Image Restoration for Effective Object Detection in Adverse Weather

**DOI:** 10.3390/s24196330

**Published:** 2024-09-30

**Authors:** Xiaochen Huang, Xiaofeng Wang, Qizhi Teng, Xiaohai He, Honggang Chen

**Affiliations:** 1 College of Electronics and Information Engineering, Sichuan University, Chengdu 610065, China; 2 Key Laboratory of Computer Vision and System, Ministry of Education, Tianjin University of Technology, Tianjin 300384, China; 3 Yunnan Key Laboratory of Software Engineering, Yunnan University, Kunming 650600, China

**Keywords:** object detection in various weather scenes, restoration-assisted object detection, degradation type awareness, multi-task joint learning

## Abstract

Despite significant advancements in CNN-based object detection technology, adverse weather conditions can disrupt imaging sensors’ ability to capture clear images, thereby adversely impacting detection accuracy. Mainstream algorithms for adverse weather object detection enhance detection performance through image restoration methods. Nevertheless, the majority of these approaches are designed for a specific degradation scenario, making it difficult to adapt to diverse weather conditions. To cope with this issue, we put forward a degradation type-aware restoration-assisted object detection network, dubbed DTRDNet. It contains an object detection network with a shared feature encoder (SFE) and object detection decoder, a degradation discrimination image restoration decoder (DDIR), and a degradation category predictor (DCP). In the training phase, we jointly optimize the whole framework on a mixed weather dataset, including degraded images and clean images. Specifically, the degradation type information is incorporated in our DDIR to avoid the interaction between clean images and the restoration module. Furthermore, the DCP makes the SFE possess degradation category awareness ability, enhancing the detector’s adaptability to diverse weather conditions and enabling it to furnish requisite environmental information as required. Both the DCP and the DDIR can be removed according to requirement in the inference stage to retain the real-time performance of the detection algorithm. Extensive experiments on clear, hazy, rainy, and snowy images demonstrate that our DTRDNet outperforms advanced object detection algorithms, achieving an average mAP of 79.38% across the four weather test sets.

## 1. Introduction

Object detection technology, which is dedicated to extracting target information such as spatial coordinates, semantic category, and physical dimensions from images or videos, constitutes one of the foundational tasks in the field of computer vision. In recent years, the rapid evolution of deep learning has catalyzed significant advancements in learning-based object detection technologies, showcasing exceptional accuracy and real-time performance across diverse datasets sourced from various sensors, including cameras, surveillance systems, vehicles, drones, and satellites [1,2,3,4,5]. Consequently, this technology is increasingly being utilized in various domains, including autonomous driving [6], environmental monitoring [7], military reconnaissance [8], and ship fire detection [9]. Nevertheless, mainstream detection algorithms are generally optimized and assessed using high-quality datasets such as MS COCO [10] and PASCAL VOC [11]. The data captured by sensors under adverse weather conditions frequently lead to degraded images characterized by poor visibility and blurred target details, posing significant challenges for the detection model in extracting target features. This constrains the model’s generalization and robustness, resulting in a serious decline in detection performance under adverse weather conditions such as haze, rain, and snow. Therefore, enhancing the stability of object detection algorithms in these severe environments has become an urgent issue that necessitates resolution.

To address this issue, the most intuitive solution is to employ image enhancement techniques [12,13,14,15,16,17,18,19,20] as a preprocessing step to mitigate the effects of adverse weather factors. While these techniques generally improve the visual quality of images, the resulting restored outputs may not be optimally tailored for machine perception. Consequently, the performance of individually trained two-stage restoration-detection frameworks is suboptimal. Furthermore, some researchers [21,22,23] have concurrently optimized restoration and detection networks in a cascading manner to ensure that the restored outputs are more conducive to the perception of the detection network. Although these methods do enhance detection accuracy under inclement weather conditions, they inevitably introduce additional model parameters, which compromises real-time object detection.

**Figure 1:**
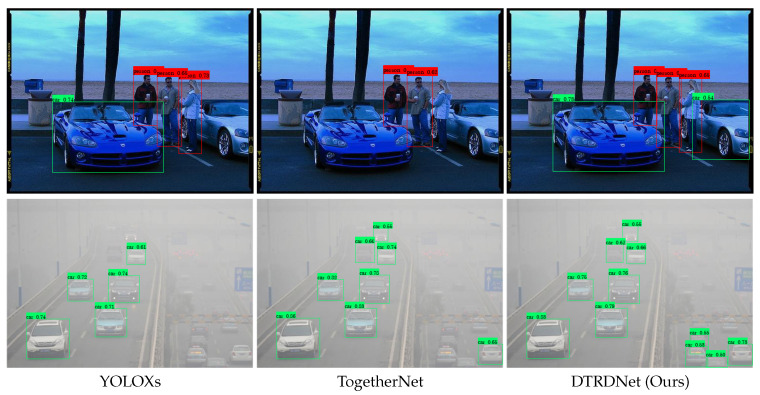
Detection results on clean and hazy images of YOLOXs [30], TogetherNet [6] and our DTRDNet. The three models are optimized for adverse weather image. The clean image is from VOC [11] and the hazy image is from RTTS [31].

Recently, several studies [23,24,25,26,27] have focused on enhancing object detection in adverse weather conditions through transfer learning, particularly employing domain adaptation methods. These approaches primarily offset the domain gap [28,29] between the source (clear images) and target domains (degraded images), facilitating improved learning from clear to degraded images. While these methods can extract intrinsic features of input images rather than relying solely on restored features, they often overlook the fact that degraded factors complicate target feature extraction and lead to an over-reliance on source domain information, which ultimately limits their performance. In contrast to the aforementioned approaches, TogetherNet [6] integrates image restoration and object detection within a multi-task joint learning framework. It employs a restoration decoder that shares features encoded by the backbone of the detection network, along with a regression loss to optimize this shared backbone. As a result, clear image features can be extracted even when inputting degraded images, significantly enhancing object detection performance for such cases. However, this method is specifically designed for certain adverse weather conditions and has several limitations that affect the stability of the detection model: (1) the network lacks awareness of varying weather conditions when processing different types of images; (2) the entire joint learning framework is trained exclusively on degraded images, neglecting more common normal weather scenarios. As presented in Figure 1, although the adverse weather image object detector TogetherNet [6] significantly improves detection accuracy for hazy images, its performance on clear images deteriorates compared to YOLOXs [30]. Therefore, further investigation is needed to design a detection model that can adapt to diverse degraded images while maintaining performance on clear images.

In this study, we introduce DTRDNet by improving the restoration module and bridging image classification [32] based on TogetherNet [6], which is designed to improve precision and robustness in object detection for images captured under diverse weather conditions, including both clear and various degraded scenarios. Firstly, to avoid excessive model complexity and the limitation of adapting to only one specific degradation type, we construct a framework based on the joint learning paradigm similar to TogetherNet [6]. This involves feeding various degraded images along with clear images into the detection model during the training stage to better accommodate diverse weather conditions, allowing the model to learn more recognizable target features from clear images. However, introducing clear images during the training phase can interfere with the restoration module, and extracting restored features from clear images may hinder their detection. To eliminate the mutual interference between clear images and the restoration network, we embed a degradation discriminator (DD) between the shared feature encoder and the restoration decoder. This discriminator distinguishes between clear and degraded images using degradation type labels, ensuring the restoration decoder only processes degraded images. Additionally, removing unnecessary restoration training of clear images offers the added benefit of reducing training computational costs. Building upon this approach, we further optimize the restoration decoder to leverage more low-level features that enhance the extraction of detailed image features, thereby improving the model’s ability to accurately position objects for detection. The combination of a DD and our optimized restoration decoder forms our degradation discrimination image restoration decoder (DDIR). Finally, we construct a degradation category predictor (DCP) to imbue the encoded features with awareness of degradation types by generating predicted degradation categories for each input during training. This enhancement improves the model’s detection stability under diverse weather conditions and can provide additional environmental information as needed. As shown in Figure 1, our method outperforms both the current detector YOLOXs [30] and the adverse weather detector TogetherNet [6] in detecting both degraded and clear images.

Previous studies have predominantly focused on addressing specific adverse weather conditions, which is insufficient for effectively handling multiple weather scenarios with a single model. As a result, practical applications often require the deployment of multiple models to accommodate diverse weather conditions, incurring substantial costs. Our research aims to tackle the challenge of object detection under various complex weather conditions through a unified network. The proposed approach significantly enhances the accuracy and stability of the detector across different weather scenarios without incurring additional inference costs.

On the whole, our study makes the following contributions:

- On the basis of TogetherNet, we further develop the multi-task joint learning paradigm by bridging the tasks of detection, restoration, and classification to enhance the performance and stability of the detector in different environments.
- The proposed DDIR effectively mitigates the influence of clear data on the restoration model and improves the restoration decoder to bolster the model’s capacity to extract detailed features. This facilitates the model to more effectively encode clear features for both clean and degraded images.
- The introduced DCP endows the feature extraction network with degradation category awareness, thereby enhancing the model’s stability under varying weather conditions.

## 2. Related Work

### 2.1. Object Detection

Object detection is one of the most important tasks in the computer vision field, widely employed in domains such as ship detection [9,33], autonomous driving [34], and military surveillance [8]. Recently, with the rapid advancements in deep learning, learning-based detectors have emerged as the mainstream approach for both application and research. These can be broadly categorized into region proposal-based methods and regression-based methods. The regional proposal-based approach first generates the candidate proposal ARES by methods such as selective search and edge boxes, and then performs adjustments using the subsequent detection model. The most representative proposal-based detector, R-CNN [35], first generates proposal areas through a convolutional neural network and then uses a support vector machine (SVM) to classify them. Inspired by the success of R-CNN in object detection, a large number of variants based on this framework have been developed, each aiming to enhance the original model’s performance and address its limitations. These variants include Fast R-CNN [36], Faster R-CNN [37], Libra R-CNN [38], Dynamic R-CNN [39], and Mask R-CNN [40], among others. While the regional proposal-based approaches generally exhibit certain advantages in accuracy, they often come with higher computational costs and perform worse in terms of detection speed because the requirements for localization and classification are handled in two stages.

In contrast to the regional proposal-based approach, regression-based approaches represented by the YOLO series [5,8,30,41,42,43,44,45], SSD [46], RetinaNet [47], and CenterNet [48], among others, complete the prediction of target categories and localization in a single stage. This type of detector usually utilizes a feature encoder to extract image features and then decodes the features to predict the category and location information of targets. It should be noted that regression-based detection algorithms typically preset anchor boxes with different aspect ratios, which saves time and computational resources required to generate proposal boxes compared to regional proposal-based detectors, thus achieving faster detection speeds. In addition, the two optimizations of Focal Loss (FL) [47], used to mitigate the problem of positive and negative sample imbalance during the training stage, and Feature Pyramid Networks (FPN) [49], employed to address the issue of variation in target sizes, greatly improve the detection performance of regression-based single-stage detectors, making them better suited to satisfy the trade-off between performance and speed. In summary, although the performance of regression-based single-stage object detection algorithms is not as accurate as that of two-stage algorithms, they do not require generating proposal boxes and can directly output information about object categories, positions, and confidence in an end-to-end regression method. This provides these methods with a significant advantage in real-time performance.

### 2.2. Object Detection in Adverse Weather Conditions

The advancement of learning-based object detection technology has led to its widespread application across various scenarios. Nevertheless, conventional detection algorithms are susceptible to weather degradation, which poses challenges when encountering adverse environments. Although an increasing number of studies have concentrated on object detection in low-quality images, research specifically addressing this area remains limited.

Early algorithms for object detection in low-quality images predominantly employ image restoration techniques, including dehazing [12,13,14,50], deraining [14,16,21,51], and desnowing [17,18], among others. These methods function as preprocessing steps designed to restore degraded images before the execution of object detection. Intuitively, clearer images obtained from restoration algorithms should enhance detection performance. However, empirical evidence suggests that even when the clarity of restored images closely resembles that of real clear images, their detection performance may not improve. This discrepancy could be attributed to a domain gap between the restored images and actual clear images. Furthermore, some researchers have sought to integrate image restoration and object detection networks to alleviate the adverse effects of degraded information.

Sindagi et al. [23] improve the object in rainy and foggy weather by an unsupervised domain-adaptive framework based on priors. Liu et al. [24] develop an image-adaptive network for object detection in adverse weather conditions, which integrates restoration and detection models in an end-to-end framework that can learn appropriate parameters to enhance images for detection in a weakly supervised manner. Wang et al. [6] employ a unified joint learning paradigm to integrate image restoration and object detection, training a shared feature encoder for the two tasks and jointly optimizing them through restoration and detection losses, thereby enhancing detection performance under adverse weather conditions. Wang et al. [52] employ degradation modeling for feature transformation in the object detection task. Furthermore, several methods [53,54,55,56] have been proposed to address the issue of degradation distraction through a domain adaptation approach. For instance, Zhang et al. [56] introduce a domain-adaptive YOLO framework to enhance the capabilities of single-stage detectors for transfer across different domains.

Although some progress has been achieved in existing object detection algorithms for adverse weather conditions, there are still some shortcomings. For instance, most optimization methods for object detection in adverse weather conditions do not distinguish the clear and various degraded images, resulting in approaches that are tailored exclusively to specific degradations, which can even decrease the detection performance of clear images. Therefore, we further investigate the existing challenges in object detection algorithms for multiple weather conditions. Our objective is to develop a detection framework capable of leveraging degraded information to distinguish between various degradation types, thereby addressing multiple weather degradations simultaneously while maintaining effective detection on clear images.

## 3. Methods

**Figure 2:**
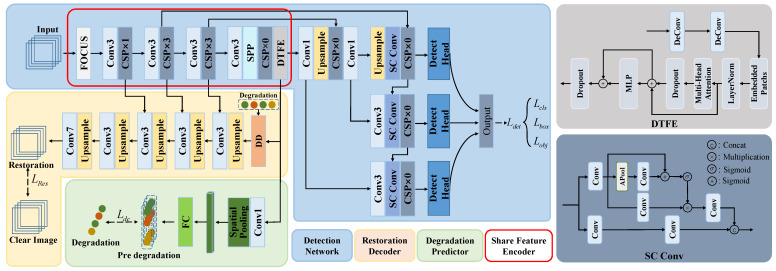
The framework of our proposed DTRDNet. There are three parts: (1) the object detection network based on YOLOX that can be divided into the shared feature encoder (SFE) and the objection detection decoder, (2) our proposed restoration decoder (DDIR), and (3) the degradation predictor (DCP). The DD is our proposed degradation discriminator, and its input, “Degradation”, is the practical degradation type of image. The DDIR and the DCP can be selectively removed in the inference stage if that specific function is not needed. DTFE refers to Dynamic Transformer Feature Enhancement module [6], and SC Conv represents self-calibrated convolutions [57]. “APool” is the average pooling operation.

Degraded images caused by adverse weather, such as haze, rain, and snow, significantly decrease the performance of object detection and pose a considerable challenge to the environmental perception capabilities of detectors. To enhance the robustness of object detection models under various weather conditions, we propose DTRDNet based on TogetherNet [6]. It first employs a multi-task joint learning paradigm to optimize both detection and restoration tasks simultaneously for diverse weather images. Additionally, we embed a degradation discriminator (DD) between the feature encoder and restoration decoder to form the Degradation Discrimination Image Restoration Decoder (DDIR), aiming to mitigate the interference of clear images on the restoration module by leveraging degradation type information. On this basis, we propose a degradation category predictor (DCP), which utilizes degradation information as supervision to enhance environmental perception capability. The overview of the proposed framework is illustrated in Figure 2. Next, we will specify our algorithm in detail.

### 3.1. Overall Architecture

As illustrated in Figure 2, our proposed DTRDNet integrates three tasks: object detection, restoration, and classification. It primarily consists of three components: (1) an object detection network that incorporates a shared feature encoder (SFE) and an object detection decoder, (2) our proposed restoration decoder (DDIR), and (3) a degradation predictor (DCP). For convenience, we first introduce the overall workflow of our algorithm.

Differing with current optimization algorithms for adverse weather image object detection, we are devoted to utilizing restoration and degradation discrimination to improve detection performance for degraded images while retaining performance on clear images. For this reason, we mix the clear and degraded images together to optimize the network parameter during the training stage. The input is first processed by the SFE to extract multi-scale image features and learn degradation category information. Subsequently, the encoded features are fed into the object detection decoder, the DDIR, and the DCP to generate the detection results, restored clear images, and predicted degradation types. Finally, we formulate detection, restoration and degradation classification loss functions to jointly optimize the whole framework. This enables the SFE to distill knowledge from restoration and degradation classification tasks to assist with the detection task. Specifically, the DDIR enables the SFE to extract clear multi-scale features regardless of whether the input is a clear or degraded image, thereby improving detection performance under both clean and degraded weather conditions. The DIP endows the SFE with the capability to discern degradation type information within images, thereby enhancing stability when encountering various weather-induced degradations. Notably, during the inference phase, it is feasible to selectively omit the DDIR and the DCP to mitigate computational complexity without compromising object detection accuracy.

### 3.2. Object Detection Network

Although the aforementioned deficiencies require further attention, it is unequivocal that TogetherNet [6] represents a significant advancement in the detection of adverse weather conditions. In particular, its baseline network, YOLOXs, serves as a prominent regression-based detector, and the enhancements associated with DTFE and SC Conv have demonstrated considerable efficacy in object detection. Consequently, we retain its detection architecture, which comprises a backbone network and a detection head. The backbone operates as an SFE module to encode multi-scale image features for subsequent decoding modules, while the detection head functions as the object detection decoder within our framework.

#### 3.2.1. Shared Feature Encoder

Given a clear or degraded input image $X\in R^{3\times 640\times 640}$, the focus first divides the image into four patches and concatenates them to diminish spatial dimensions, thereby contributing to reduced computational costs. Then, a series of cascaded convolutions and CSP (Cross Stage Partial Network) are utilized to extract multi-scale features. In this process, SPP and DTFE are employed to enhance the feature representation capability of the entire encoder. There are four scale features extracted by the SFE, which can be mathematically expressed as follows:

(1) $$f_{\times 4},f_{\times 8},f_{\times 16},f_{\times 32}=E_{SFE}\left(X\right),$$

where $E_{SFE}\left(\;\cdot \;\right)$ denotes the encoding process of the SFE, and $f_{\times 4},f_{\times 8},f_{\times 16},$ and $f_{\times 32}$ represent encoded image features at different scales, with subscript ×n indicating the size difference relative to the input image. Specifically, $f_{\times 4}\in R^{64\times 160\times 160}$, $f_{\times 8}\in R^{128\times 80\times 80}$, $f_{\times 16}\in R^{256\times 40\times 40}$, and $f_{\times 32}\in R^{512\times 20\times 20}$. These multi-scale features will be utilized by the object detection decoder (DDIR) and the DCP to generate results for object detection, image restoration, and degradation category prediction. Consequently, for object detection tasks, the optimized SFE following backpropagation ensures that these features contain information from clear images and awareness of degradation categories. This significantly enhances feature extraction capabilities and improves generalization across diverse environments.

#### 3.2.2. Object Detection Decoder

As previously mentioned, we employ the head of TogetherNet [6] as our object detection decoder. It includes a neck and detect head that have been enhanced by self-calibrated convolutions (SC Conv) [57]. The neck consists of a widely used feature pyramid network (FPN) [49] and a path aggregation network (PANet) [58] structure, which integrate high-level semantic features with low-level texture features to generate spatially scaled features for targets of varying sizes. Specifically, the FPN upsamples the high-level feature after adjusting its channel number using a 1×1 convolution, and then concatenates it with the corresponding scale’s feature from the SFE. This operation is repeated twice to produce two fused features with different scales. The formulary description is as follows:

(2) $$f_{\times 32}^{FPN}=Conv_{1,1}\left(f_{\times 32}\right),$$

(3) $$f_{\times 16}^{FPN}=Conv_{1,1}\left(CSP\left(Cat{\left[Up\left(f_{\times 32}^{FPN}\right),f_{\times 16}\right]}_{c}\right)\right),$$

(4) $$f_{\times 8}^{FPN}=CSP{\left(Cat\left[SC\left(Up\left(f_{\times 16}^{FPN}\right)\right),f_{\times 8}\right)\right]}_{c}),$$

where $Conv_{k,s}\left(\;\cdot \;\right)$ denotes convolution with a kernel size of k×k and a stride of *s*. The operation Up(·) signifies double upsampling. The notation $Cat{\left[\;\cdot \;,\;\cdot \;\right]}_{c}$ represents the concatenation operation along the channel dimension. CSP(·) and SC(·) denote the functions of CSP and SC modules, respectively. Followed by the PANet, it convolves the feature map $f_{\times 8}^{FPN}$ to generate two smaller-sized features, $f_{\times 16}^{PAN}$ and $f_{\times 32}^{PAN}$, as formulated below:

(5) $$f_{\times 16}^{PAN}=CSP\left(Cat{\left[SC\left(Conv_{3,2}\left(f_{\times 8}^{FPN}\right)\right),f_{\times 16}^{FPN}\right]}_{c}\right);$$

(6) $$f_{\times 32}^{PAN}=CSP\left(Cat{\left[SC\left(Conv_{3,2}\left(f_{\times 16}^{PAN}\right)\right),f_{\times 32}^{FPN}\right]}_{c}\right).$$

In the aforementioned FPN and PANet, self-calibrated convolutions and CSP are employed to expand the receptive field and further enhance features, which are subsequently decoupled for improved object detection. Ultimately, we utilize three detection heads to separate the features $f_{\times 8}^{FPN}$, $f_{\times 16}^{PAN}$, and $f_{\times 32}^{PAN}$ in order to output the detection results.

### 3.3. Restoration Network

The most current restoration-based adverse weather object detection algorithms focus solely on restoring clear images from single degraded inputs, which inadequately addresses the detection requirements across various scenarios when employing a single model. Furthermore, the utilization of multiple detection models tailored for various environments considerably complicates the deployment process. To enhance detection performance across diverse degraded images while ensuring effectiveness for clear images, we propose incorporating degradation type information to effectively differentiate between clear and degraded images. Subsequently, we optimize the image restoration decoder to establish the DDIR. It is designed to exclusively restore degraded images, thereby eliminating interference from clear images in the restoration process.

**Figure 3:**
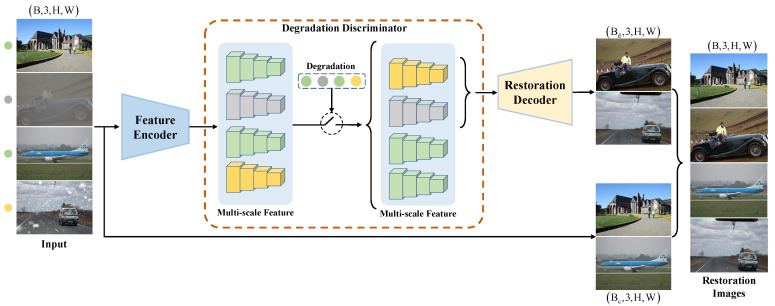
The restoration process of our DTRDNet. It can be seen as an encode–decode structure with the proposed degradation discriminator (DD) between encoder and decoder. The feature encoder adopts the SFE. Restoration decoder and DD make up our DDIR. $B_{d}$ and $B_{c}$ refer to the number of degraded and clear images of a batch of input.

Figure 3 depicts the comprehensive restoration process of our DTRDNet. We integrate our proposed DD between the SFE and the restoration decoder to determine whether an input is a degraded image. For a batch of inputs, regardless of their classification as degraded or clear images, the SFE first extracts image features, while the DD utilizes degradation type labels to assess the degradation status of each image. If an image is identified as a degraded image, its multi-scale features extracted by the SFE are forwarded to the restoration decoder to generate a restored output. Otherwise, if the input is a clear image, we do not process the corresponding features with the restoration decoder; instead, we utilize the clear input image as the output result. All restored degraded images and clear images are concatenated to generate the final restoration output. It should be noted that, to maintain the original order of corresponding images within a batch for calculating restoration loss, each image in the same batch is processed one by one during this procedure, which also helps alleviate the training burden. Specifically, as depicted in Figure 2, for a degraded image, our DDIR utilizes its multi-scale features $f_{\times 4},f_{\times 8},f_{\times 16}$, and $f_{\times 32}$ extracted by the SFE to generate restored images. The high-level feature $f_{\times 32}$ is restored to match input size through five cascading upsampling and convolution operations. In this process, lower-level features $f_{\times 16},f_{\times 8}$, and $f_{\times 4}$ are progressively integrated. The process can be expressed as follows:

(7) $$f_{\times 16}^{R}=Conv_{3,2}\left(Up\left(f_{\times 32}^{D}\right)\right),$$

(8) $$f_{\times 8}^{R}=Conv_{3,2}\left(Up\left(Cat{\left[f_{\times 16}^{R},f_{\times 16}\right]}_{c}\right)\right),$$

(9) $$f_{\times 4}^{R}=Conv_{3,2}\left(Up\left(Cat{\left[f_{\times 8}^{R},f_{\times 8}\right]}_{c}\right)\right),$$

(10) $$R^{D}=Conv_{3,2}\left(Up\left(Conv_{3,2}\left(Up\left(f_{\times 4}^{R}\right)\right)\right)\right),$$

where $f_{\times 32}^{D}$ refers to the $f_{\times 32}$ of degraded images, $f^{R}$ denotes the feature produced by the DDIR, and $R^{D}\in R^{3\times H\times W}$ stands for the output of restoration decoder, with *H* and *W* denoting the spatial dimensions of input images. For inputs in a batch without degradation, we concatenate it with restored output $R^{D}$ of the degraded images to form the final restoration results as follows:

(11) $$R=Cat{\left[R^{D},X^{C}\right]}_{b},$$

where $X^{C}$ refers to the clear image contained in inputs, *R* represents the final restoration result, and $Cat{\left[\;\cdot \;,\;\cdot \;\right]}_{b}$ denotes the operation of concatenation in the order of input batch.

### 3.4. Degradation Category Predictor

We incorporate degradation type information into the DDIR to bypass the process of restoring clear images, thereby mitigating its impact on model parameter updating. The primary objective remains extracting clear image features from degraded images. However, in reality, the features of a specific target vary under different weather conditions. This insight motivates us to recognize that for training a detection model adapting to various weather conditions, the model must possess weather awareness capabilities. Consequently, we employ degradation type information as labels to train a degradation category classifier capable of predicting the weather in input images, referred to as DCP.

As exhibited in Figure 2, the DCP leverages the features of the SFE to predict the degradation category, aiming to avoid increased model complexity and learn degradation category awareness of the SFE to facilitate object detection in diverse weather conditions. The DCP incorporates a simple classifier that consists of a convolution layer with a 1×1 kernel size for adjusting the number of feature channels, a spatial pooling layer (Pool) for extracting global features for classification, and a fully connected layer (FC) for generating prediction results. The predicted degradation category ($O^{D}$) of the DCP can be formulated as follows:

(12) $$O^{D}=FC\left(Pool\left(Conv_{1,1}\left(f_{\times 32}\right)\right)\right).$$

### 3.5. Loss Function

To jointly train the entire framework for object detection, image restoration, and degradation classification, we introduce three distinct loss functions corresponding to the output of each decoder. Specific details are provided below.

**Object Detection Loss:** We evaluate the disparity between the object detection results and their corresponding labels to compute the detection loss, which consists of three components: (1) $L_{box}$ for the positional variance between predicted and actual bounding boxes, (2) $L_{cls}$ for target category prediction, and (3) $L_{obj}$ for bounding box confidence. This can be expressed as follows:

(13) $$L_{Det}=\lambda L_{box}+L_{cls}+L_{obj},$$

where the weight λ for $L_{box}$ is set to 5. The losses $L_{box}$ and $L_{cls}$ are computed using IOU loss and cross-entropy function, respectively. The loss function $L_{obj}$ adopts focus loss [47] to address the issue of imbalanced positive and negative samples in object detection tasks.

**Image Restoration Loss:** To acquire information for image restoration, we compute the mean square error (MSE) between the input and restoration result as our restoration loss $L_{Res}$. It is important to note that, since both clear images and restored degraded images may be present in the final restoration result, the MSE of the corresponding clear image in the input *X* and restoration result *R* is zero. Therefore, the restoration loss $L_{Res}$ can be represented as follows:

(14) $$L_{Res}=\frac{1}{N}\sum_{i=1}^{N}{∥X-R∥}_{2}=\frac{1}{N_{D}}\sum_{i=1}^{N_{D}}{∥X_{C}^{D}-R^{D}∥}_{2},$$

where *N* is the batch size of input. $N_{D}$ refers to the number of degraded images in a batch. $R^{D}$ and $X_{C}^{D}$ represent the restored degraded image and its corresponding clear image target, respectively.

**Degradation Classification Loss:** To make the network possess degradation category awareness, we introduce the cross-entropy function as the degradation classification loss $L_{dc}$ to assess whether the predicted results of DIP are consistent with the actual label, expressed as

(15) $$L_{dc}=-\sum_{i=1}^{K}y_{i}\log\left(O_{i}^{D}\right),y_{i}=\left\{\begin{array}{cc} 1 & i=t\\ 0 & otherwise, \end{array}\right.$$

where *K* is set to 4, representing the degradation conditions of images, including clean, hazy, rainy, and snowy. *t* refers to the true label of degradation type.

Based on the above definition, the complete loss of our DTRDNet is formulated as

(16) $$L=\alpha L_{Det}+\beta L_{Res}+\gamma L_{dc},$$

where α, β, and γ are the balance coefficients for object detection loss, restoration loss, and degradation classification loss, respectively. We follow the set of TogetherNet [6] with α=0.2 and β=0.8. The γ is set to 0.01. Based on the principle of back propagation, the parameters of the three decoders will be updated according to their respective loss functions to produce the desired output. The SFE will be optimized using three simultaneous loss functions, allowing it to extract and encode information for object detection, image restoration, and degradation classification. The optimization for image restoration ensures that the SFE can extract clear image features, even from degraded input images. Similarly, the regression for predicting degradation categories endows the shared feature with awareness of different degradation types. Both clear image features and degradation category awareness contribute to the performance and robustness of the detector under various weather conditions.

## 4. Experiment and Results

This section presents a comprehensive analysis of our experiments. We commence by detailing the experimental setup, which includes datasets and implementation specifics. Subsequently, we provide both quantitative and qualitative evaluations of our proposed DTRDNet, comparing its performance against other state-of-the-art object detection algorithms. Finally, we conduct an ablation experiment to validate the efficacy of our improvements.

### 4.1. Datasets

**Train and Test Dataset. (1) VOC-FOG.** This is a synthesized hazy dataset derived from the VOC dataset [11] proposed by TogetherNet [6], comprising a total of 11,707 hazy images across five object categories (namely, car, bus, motorbike, and person), with 9578 images designated for training (VOC-FOG-train) and 2129 for testing (VOC-FOG-test).

**Train and Test Dataset. (2) VOC-Clean.** We select the images utilized to synthesize the VOC-FOG dataset to constitute a clear image dataset, ensuring that it encompasses the same object categories and number of images as VOC-FOG. The training set, comprising 9578 images (VOC-Clean-train), is integrated with the degraded image training set for model training. The performance of the detector on clear images is assessed using its testing set (VOC-Clean-test), which consists of 2129 images.

**Train and Test Dataset. (3) VOC-Rain.** Similar to VOC-FOG, we employed the rain streak synthesis method of RainDS [59] to synthesize rainy images using the images from VOC-Clean. Furthermore, in order to more accurately simulate the effects of rainy days on imaging results, we synthesized various types of rain streaks with different orientations and intensities. The synthesized rainy images are divided into a training set (VOC-Rain-train) comprising 9578 images and a testing set (VOC-Rain-test) comprising 2129 images.

**Train and Test Dataset. (4) VOC-Snow.** We generate snowy images by combining high-quality images from the VOC-Clean dataset with snow masks from the CSD dataset [60]. The snow masks are applied to the high-quality images using a random weight ranging from 0.5 to 1.0, simulating varying intensities of snow. Subsequently, we create a snowy dataset (VOC-Snow) comprising 9578 training images (VOC-Snow-train) and 2129 testing images (VOC-Snow-test). Similarly, both the training and testing sets consist of five classes: car, bus, motorbike, bicycle, and person.

**Real Scenario Test Dataset. (1) RTTS [31].** Due to the difficulty of capturing paired hazy-clean images in real-world scenarios, RTTS was proposed to evaluate the performance of dehazing algorithms from a task-driven perspective. This is a comprehensive dataset for object detection in real-world hazy scenes, containing 4322 hazy images annotated with five object categories (car, bus, motorbike, and person) corresponding to those in the training set.

**Real Scenario Test Datasets. (2) Foggy Driving dataset [61].** This dataset provides a practical repository of fog-affected scene images tailor-made for applications in object detection and semantic segmentation, and contains a total of 101 authentic fog-affected scene images annotated with 466 vehicle instances (including car, bus, train, truck, bicycle, and motorcycle) and 269 human instances. It is noteworthy that the dataset includes eight different categories of annotated instances, with only five of these categories selected for testing to maintain consistency with the training set.

### 4.2. Implementation Details

The implementation of this paper is conducted on the Ubuntu operating system using an NVIDIA 3090 GPU and the PyTorch framework. During training, we employ stochastic gradient descent (SGD) as the optimizer to update the model parameters. The initial learning rate is set to 1 × 10^−2^, with a momentum of 0.937 and a weight decay of 5 × 10^−4^. Additionally, we utilize a cosine annealing strategy [62] to reduce the learning rate for improved convergence. It is important to emphasize that the inclusion of various categories of degraded images will lead to a multiplicative increase in data volume. The utilization of a wider range of degradation types for training will result in an increase in the times of parameter updates. Consequently, to ensure fairness, different quantities of training datasets will be associated with diverse epoch settings in subsequent experiments. In terms of input, the spatial dimensions of all images are resized to 640 × 640 during both training and testing stages, with a fixed batch size of eight. Additionally, since mosaic data augmentation can disrupt the degradation type of the input and complicate the training of the restoration network, we close mosaic augmentation during the training phase. In contrast to universal detection algorithms, our proposed method employs clear images to supervise the training of the restoration branch. Consequently, we apply identical data enhancement to the input image and its corresponding clear image, including hue saturation value (HSV), random scaling, translation, and flipping. The augmented clear images are used as targets for restoration loss.

To comprehensively assess the performance of our algorithm across various weather conditions, including clear and adverse scenarios such as haze, rain, and snow, we conduct separate implementations for single degradation weather and diverse weather conditions. We then compare these results with those obtained from other advanced adverse weather image detection models.

### 4.3. Implementation Results

We assess the detection performance of our DTRDNet using the widely adopted mean average precision (mAP) metric across multiple target categories, with an intersection over union (IOU) set to 0.5. The comprehensive investigation are conducted under both single and multiple degradation conditions, with the results summarized in the table below. In the tables presented in this paper, the best results are highlighted in bold, while the second-best results are are indicated by underlining. The following section presents comparative experiments of our proposed method in these scenarios, with our ablation experiments provided at the end.

#### 4.3.1. Hazy Scene Object Detection

To assess the performance of the proposed DTRDNet in hazy conditions, we conducted a comparative analysis with other state-of-the-art object detection algorithms designed for adverse weather scenarios. Generally, these comparative methods can be categorized into three parts. (i) The first part includes the common “object detection (OD)” YOLOXs models as baselines, which are trained on degraded images (VOC-FOG-train) and clear images (VOC-Clean-train), respectively, denoted as YOLOXs and YOLOXs*. (ii) The second part is the “IR + OD” approach, which prepossess the hazy images by the dehazing algorithms to produce clear images, and then detects the clear images using the YOLOXs [30] model trained on VOC-Clean-train. Five mainstream image dehazing algorithms are selected for this purpose, incorporating DCP [12], AODNet [63], FFANet [50], AECRNet [13], and RestorNet [14]. We combine these methods with YOLOXs* [30] to form “IR + OD” type algorithms, named DCP-YOLOXs*, AODNet-YOLOXs*, FFANet-YOLOXs*, AECRNet-YOLOXs*, and RestorNet-YOLOXs*, respectively. It is worth noting that all the dehazing algorithms are trained on the OTS dataset from the RESIDE [31] dataset to ensure data consistency with RTTS [31]. (iii) The third part refers to the “Object Detection in Adverse Weather (ODAW)” algorithms that do not require the use of restoration algorithms or other preprocessing steps, including DS-Net [64], IA-YOLO [24], and TogetherNet [6]. To ensure a fair comparison, we retrained the aforementioned “ODAW” algorithms using the VOC-FOG-training dataset. As our framework requires information on different degradation types, we combine the VOC-Clean-train and VOC-FOG-train datasets to form our training set for hazy image object detection. We train the model for 50 epochs to mitigate overfitting that may arise from doubling our training images, while other methods are trained for 100 epochs. Subsequent experiments also adjust the number of training epochs based on the combination of datasets.

**Object Detection Results on Synthetic Hazy Image Dataset.** We first employ the synthetic dataset VOC-FOG-test to validate the performance of the proposed DTRDNet and the comparative algorithms. The results are presented in Table 1. It is evident that our proposed DTRDNet achieves a mAP of 79.86%, which is 1.72% higher than the recently advanced TogetherNet [6] dedicated to foggy object detection, with all categories achieving either the highest or second-highest average accuracy. Overall, our algorithm demonstrates superior detection performance and narrows the accuracy gap across different object classes.

**Object Detection Results on Real Scene Hazy Image Dataset.** To demonstrate the effectiveness of our approach in real-world hazy conditions, we further conduct comparative experiments on the Foggy Driving dataset [61] and the RTTS dataset [31]. As shown in Table 2 and Table 3, our method achieved the highest accuracy on both datasets. Specifically, for the Foggy Driving dataset [61], we observed an increase of 2.77% in mAP compared to YOLOXs and a 5.75% improvement over TogetherNet [6]. For the RTTS dataset, our algorithms also demonstrated significant improvements. Compared to YOLOXs [30] and TogetherNet [6], there are increases of 5.64% and 6.06% in mAP metrics, respectively. This significant improvement on the test set of real scenarios proves that our method can effectively mitigate overfitting.

Subsequently, we performed a joint analysis based on the findings presented in Table 1, Table 2 and Table 3. It is evident from Table 1 that models trained on clear images exhibit limited adaptability to degraded images when comparing YOLOXs and YOLOXs*. Furthermore, the “IR + OD” algorithms demonstrate some improvement in detection performance for YOLOXs* under general haze conditions. However, the results depicted in Table 2 and Table 3 indicate that the efficacy of “IR + OD” algorithms is notably constrained when applied to real-world hazy images. This limitation may be attributed to a domain gap between synthetic hazy datasets used for dehazing model training and real-world hazy images, resulting in limited or adverse effects of the “IR + OD” method on actual hazy weather detection performance. In contrast, our algorithm outperforms the “OD”, “IR + OD”, and “ODAW” methods under single haze degradation conditions, demonstrating superior detection performance across both synthetic and real-world hazy scenes. Notably, our approach exhibits particularly obvious improvements in authentic foggy images, indicating our method has excellent generalization in real scenarios.

**Figure 4:**
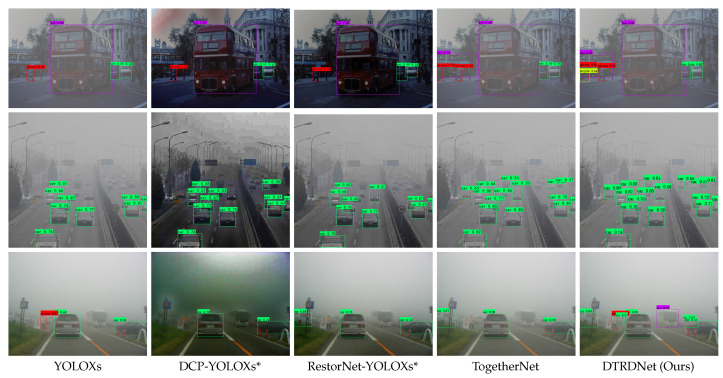
Visualization of object detection results of hazy images on both synthetic and real-world datasets. We show the images from the VOC-FOG-test [6], RTTS [31], and Foggy Driving datasets [61], respectively, from first to third row.

In addition to the above quantitative analysis, we also present the visual detection results of comparative algorithms on the VOC-FOG-test [11], Foggy Driving [61], and RTTS [31] datasets in Figure 4. The detection results show that our method can correctly detect more targets with higher confidence, further testifying to the superiority of the proposed algorithm in both synthetic hazy images and real scene hazy images compared to the comparative algorithms.

#### 4.3.2. Rainy Scene Object Detection

To evaluate the performance of the proposed DTRDNet in rainy image object detection, we conduct a comparative experiment using synthetic rainy images. Similarly to hazy scene object detection, we combine VOC-Clean-train and VOC-Rain-train as the training set to optimize our proposed model over 50 epochs for rainy object detection, while the comparative algorithms are trained on VOC-Rain-train for 100 epochs. We select YOLOXs trained on hazy images and YOLOXs* trained on clear images as the object detection (OD) methods. For IR + OD, we employ the restoration algorithms AirNet [51] and RestorNet [14] to preprocess the rainy images before object detection, titled the AirNet-YOLOXs* and RestorNet-YOLOXs*. For ODAW comparison, TogetherNet [6] is utilized for comparison. The results of these aforementioned object detection algorithms on rainy images are presented in Table 4.

**Figure 5:**
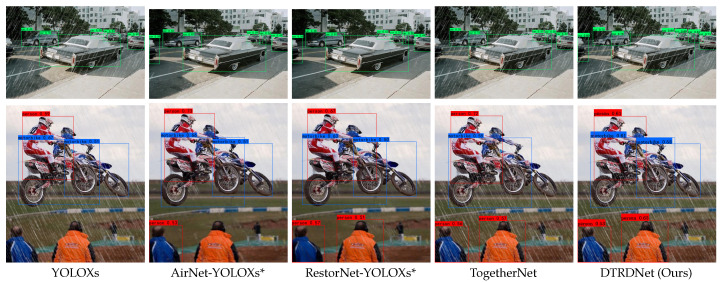
Visualization of object detection results of rainy images on VOC-Rain-test.

As shown in Table 4, our approach demonstrates superior performance across all object categories, with the exception of “Bus”, where it also achieves near-optimal accuracy. Our method outperforms the advanced TogetherNet [6], increasing mAP by 1.38%, indicating a comprehensive enhancement in model accuracy. Notably, for “Bicycle” and “Motorbike”, which exhibited lower accuracy compared to other object classes, our algorithm improves their AP by 2.69% and 3.4%, respectively, resulting in more balanced detection performance across different categories. Additionally, Figure 5 presents visual detection results in rainy conditions, demonstrating that our proposed method can accurately detect more targets with higher confidence. These results confirm the effectiveness and superiority of our proposed approach for object detection in rainy conditions.

#### 4.3.3. Snowy Scenes Object Detection

**Figure 6:**
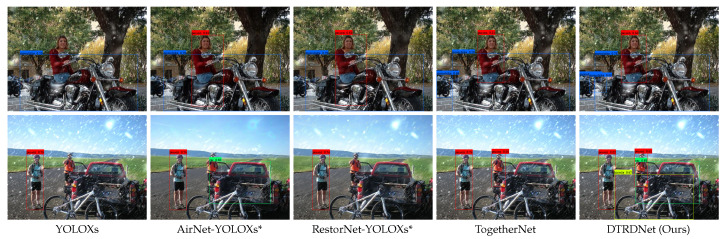
Visualization of object detection results of snow images on VOC-Snow-test dataset.

For snowy image object detection, we adopt the approach used for hazy and rainy images by leveraging the VOC-Snow-train and VOC-Clean-train datasets to train the proposed algorithms for 50 epochs, and VOC-Snow-train to train comparative algorithms for 100 epochs, followed by testing on VOC-Snow-test. The baseline method (OD) comprises YOLOXs and YOLOXs*, trained separately with snowy and clear images. In the IR + OD method, TKL [17] and LMQFormer [18] are chosen as image restoration methods for preprocessing snowy images, resulting in TKL-YOLOXs* and LMQFormer-YOLOXs*. For the ODAW method, TogetherNet is selected for comparison [6]. The specific outcomes are detailed in Table 5 and Figure 6.

According to Table 5, our DTRDNet demonstrates superior performance in the mAP metric and AP for each category compared to other methods. Specifically, the mAP shows a 2.36% increase compared to TogetherNet [6], with notable improvements in the “Motorbike” category, which exhibits a 4.56% increase in AP. Moreover, Figure 6 presents visual results of object detection on snowy images using comparative methods, further validating the effectiveness of our proposed approach. Our method excels at detecting multiple targets within complex scenes containing mixed categories and achieves higher accuracy in snow images.

#### 4.3.4. Various Weather Scenes Object Detection

**Figure 7:**
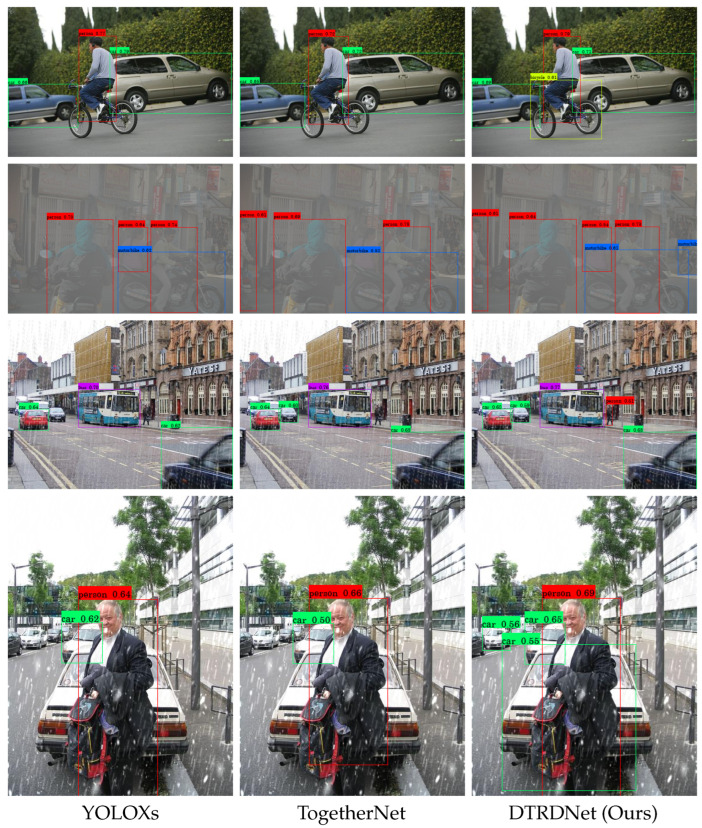
Visualization results of object detection in various weather conditions, including clean, hazy, rainy, and snowy scenes from top to bottom.

Herein above, extensive experiments have been conducted to assess the performance of the proposed DTRDNet under three adverse weather conditions: haze, rain, and snow. To further evaluate the object detection capabilities of our method across various weather conditions, we perform the experiments using a mixed dataset comprising VOC-Clean, VOC-FOG, VOC-Rain, and VOC-Snow. Specifically, the universal models are trained on the training set of the mixed dataset using our proposed method and comparison methods. The models are then tested on the testing dataset with different degradation to evaluate their performance across multiple weather conditions. Given that the “IR + OD” type is not suitable for images depicting multiple weather scenes, we exclusively employed the “OD” and “ODAW” methods for comparison. Similarly to single degradation weather image object detection, YOLOXs* was trained on clear images for 100 epochs, while YOLOXs was trained on a mixed degradation dataset. As for “ODAW”, TogetherNet [6] is chosen for comparison. It should be noted that due to an expanded dataset in this part of the experiment, we set the training epochs to 25 for models trained on a mixed dataset. Table 6 and Figure 7 present quantitative and visual detection results across four different weather conditions.

It is evident from Table 6 that the proposed method achieves superior results in object detection across various weather conditions. In comparison to TogetherNet, our method demonstrates an increase of 1.52%, 2.63%, 2.18%, and 1.77% in mAP for clean, foggy, rainy, and snowy images, respectively, resulting in an average mAP improvement of 2.03% across all four weather scenarios. Furthermore, our approach outperforms YOLOXs* in clear image object detection as well, highlighting its effectiveness in optimizing adverse weather image object detection while enhancing performance on clear images. On this basis, we further analyze the impact of introducing clear images on our method by training our model in VOC-FOG-train, VOC-Rain-train, and VOC-Snow-train, with the training epoch set to 33. The two experimental results of our DTRDNet show that the introduction of clear images has little effect on hazy and snowy images, which increases mAP by 0.03% in VOC-FOG-test and decreases by 0.01% in VOC-Snow-test. For VOC-Clean-test and VOC-Rain-test, there is an mAP boost of 0.22% and 0.13%, respectively, indicating that incorporating clear images into the training of our DTRDNet enhances detection performance for both clear and degraded images. This improvement arises because our method can better learn precise target features from clear images and eliminate interference between the clear image and the restoration module.

The visual results presented in Figure 7 illustrate the capability of our algorithm to detect multiple categories of targets under different weather conditions. In summary, our DTRDNet effectively accomplishes object detection across diverse weather conditions with a single trained model, thereby proposing a novel solution to tackle the challenges inherent in real-world object detection across diverse weather conditions.

#### 4.3.5. Ablation Studies

The above experimental results demonstrate the superiority of our method in single and multiple degraded scene object detection. Furthermore, we conducted extensive ablation experiments to evaluate the effectiveness of our optimizations over DTRDNet, analyzing each of the improvements under real degradation conditions and synthetic multiple degraded scenes.

**Effect of different components in real degraded scene.** To assess the impact of each improvements in practical application scenarios, we selected the real hazy dataset RTTS [31] as a representative. As depicted in Table 7, we first reproduce the adverse weather image object detector TogetherNet [6], which yielded a mAP of 57.53%. Subsequently, by mixing the training set with clean images from VOC-Clean-train, we optimized TogetherNet and observed an increase in mAP by 1.34%, indicating that incorporating clear images into the training set contributes to object detection under real adverse weather conditions. This may be attributed to the model can better learn target features from clear images while adapting to degraded environment within mixed data. Furthermore, introducing a DD effectively mitigates interference from clear images on the restoration branch in TogetherNet, resulting in a notable 3.11% increase in mAP. Building upon this improvement, we further improve the restoration decoder to form our DDIR, which boosts mAP by 0.71%. Lastly, integrating the DCP gives an additional 0.9% boost in mAP. The number of epochs is set to 100 for training bare TogetherNet and to 50 for subsequent stages due to mixing clean and hazy images within the training set.

**Effect of different components in synthetic multiple degradation condition.** To assess the effectiveness of our enhancements in various weather image object detection, we also perform ablation using the multiple weather dataset comprising VOC-Clean, VOC-FOG, VOC-Rain, and VOC-Snow. As shown in Table 8, compared to the baseline YOLOXs, TogetherNet [6] demonstrates an obvious improvement of 2.37% in the mAP metric. Building upon this progress, we first integrate the DD to ensure that the restoration decoder exclusively processes degraded images. This approach eliminates mutual interference between clear images and the restoration decoder, resulting in an additional 1.55% increase in mAP performance. Furthermore, the improved restoration decoder to construct the DDIR facilitates clearer image feature extraction from degraded images, leading to a further 0.28% increase in the mAP metric. Finally, incorporating a DCP to learn degradation category awareness boosts mAP by 0.19%, indicating that models equipped with degradation type awareness can achieve higher precision for object detection under various weather conditions. Moreover, as our model is capable of extracting degradation type information from images, it can also output results from the DCP to assess environmental information from images if needed, which achieves a top1 precision of 99.27% for predicting the degradation category among the four weather conditions. In terms of model complexity, all our improvements can be removed during the inference stage without imposing any additional burden when conducting only object detection tasks. If environmental information is desired alongside object detection, this would only require an additional computational cost of 0.27 G.

## 5. Limitations

**Figure 8:**
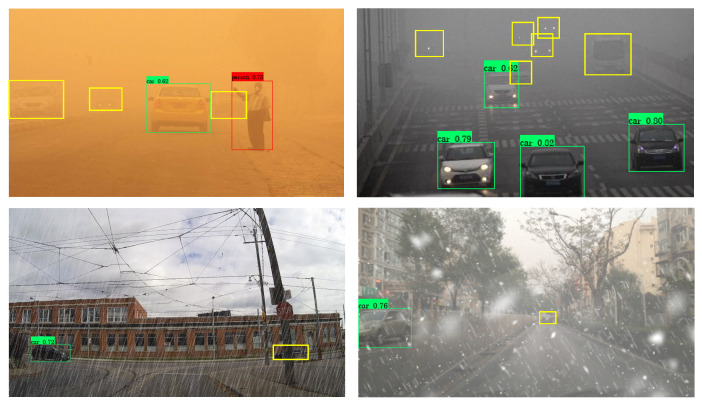
The failed case in extreme weather. The yellow boxes indicate targets that are undetected.

**Figure 9:**
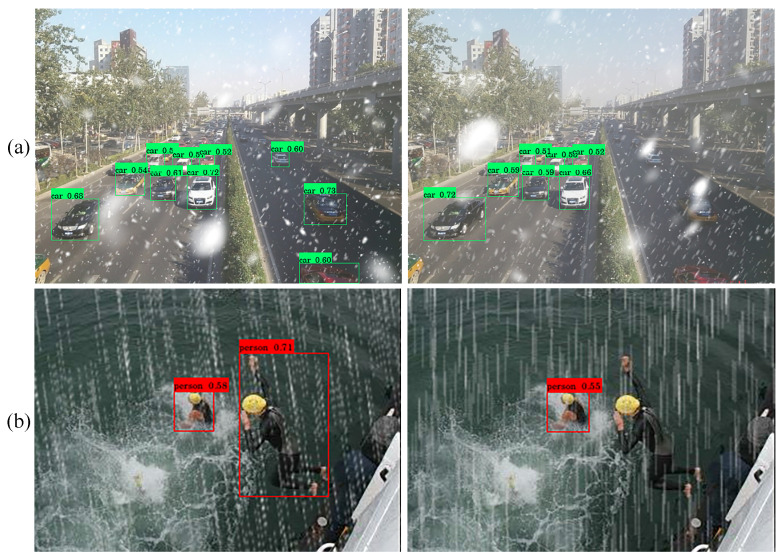
The detection performance difference caused by the varying of a certain degradation in snow and rain weather conditions. (**a**) and (**b**) refer to snow and rain images, respectively.

Although the proposed methodology leverages degradation type information to enhance detection performance across diverse weather conditions, there are still certain limitations that warrant further improvement. First, the scarcity of clear-degraded image pairs necessitates the use of synthetically degraded images for model training. While this strategy is practical, it may potentially reduce the model’s ability to adapt to real-world adverse weather scenarios. Specifically, as illustrated in Figure 8, certain targets become virtually imperceptible under extreme weather conditions, significantly complicating detection and leading to missed detections. Secondly, our approach incorporates degradation information specific to different weather types, but does not account for the varying intensities of degradation within identical weather conditions. Consequently, the proposed method falls short in assessing the severity of these conditions. This limitation is exemplified by the fluctuating detection performance illustrated in Figure 9.

## 6. Conclusions

TogetherNet [6] integrates restoration and detection tasks to enhance the detection performance of single degraded image object detection. Building upon this, our research introduces DTRDNet, which aims to address the object detection challenge under multiple complex weather conditions using a unified model. We train the model on a mixed dataset encompassing multiple weather conditions to enhance its adaptability to various weather conditions. Under these circumstances, the image restoration module designed for degraded images does not contribute to detecting clear images, and the clean images also disrupt the restoration decoder during training. Consequently, we propose a degradation discrimination image restoration decoder (DDIR) to improve the restoration branch of TogetherNet [6], introducing a degradation discriminator (DD) that leverages degradation type labels to mitigate interference between undegraded images and the restoration decoder, while further optimizing it to extract more detailed image features from degraded images. However, direct application of degradation information is insufficient; hence, we introduce a degradation category predictor (DCP) and develop a joint learning framework by integrating degradation classification with image restoration and object detection to make the feature encoder learn degradation type awareness. This approach obviously enhances object detection performance under various weather conditions. The experiment results indicate that when employing a single model for the object detection across various degraded images, our approach significantly surpass TogetherNet [6]. In the VOC-Clean, VOC-FOG, VOC-Rain, and VOC-Snow datasets, we observe improvements of 1.52%, 2.63%, 2.18%, and 1.77% in the mAP metric, respectively. In real hazy weather conditions, mAP increases by 6.06% and 5.75% on the RTTS [31] and Foggy Driving datasets [61], respectively, indicating that our method demonstrates greater applicability in actual adverse weather scenarios. Additionally, our model is capable of providing precise predictions of image degradation types at a minimal computational cost, if needed.

Our future work will focus on addressing the challenge of paired image training in unsupervised domain adaptation and evaluating the degree of degradation by incorporating text–image encoders. Additionally, we aim to enhance the performance of high-level visual tasks in real-world scenarios to better meet practical needs. 

## Figures and Tables

**Table 1:** Object detection results on the VOC-FOG-test dataset [6] of synthetic hazy images. The type of algorithms are denoted by OD (“Object Detection”), IR + OD (“Image Restoration + Object Detection”), and ODAW (“Object Detection in Adverse Weather”). “*” means the model is trained on the degradation-free images, while the methods without “*” mean they are trained on degraded images. The best results are bolded, and the sub-optimal results are indicated by underlining.

Method	Type	Class (AP (%))					mAP (%)
		Person	Bicycle	Car	Motorbike	Bus	
YOLOXs [30]	OD	67.67	**83.28**	77.75	68.91	81.70	75.86
YOLOXs* [30]	OD	73.09	57.22	69.55	59.83	77.34	67.41
DCP-YOLOXs* [12]	IR + OD	81.84	70.38	78.63	73.48	84.68	77.80
AODNet-YOLOXs* [63]	IR + OD	67.40	49.19	60.51	55.59	62.07	58.95
AECRNet-YOLOXs* [13]	IR + OD	80.47	67.82	76.97	72.46	82.73	76.09
RestorNet-YOLOXs* [14]	IR + OD	78.71	67.15	72.56	71.68	82.36	74.49
DS-Net [64]	ODAW	72.44	60.47	**81.27**	53.85	61.43	65.89
IA-YOLO [24]	ODAW	70.98	61.98	70.98	57.93	61.98	64.77
TogetherNet [6]	ODAW	84.11	69.26	79.59	72.12	**85.62**	78.14
DTRDNet (Ours)	ODAW	**84.34**	72.96	80.87	**74.83**	84.34	**79.86**

**Table 2:** Object detection results on the Foggy Driving dataset [61] of real-world hazy images. “*” means the model is trained on the degradation-free images. The best results are bolded, and the sub-optimal results are indicated by underlining.

Method	Type	Class (AP (%))					mAP (%)
		Person	Bicycle	Car	Motorbike	Bus	
YOLOXs [30]	OD	24.37	22.33	55.57	**14.29**	37.34	30.78
YOLOXs* [30]	OD	21.48	18.84	54.67	1.59	30.33	25.38
DCP-YOLOXs* [12]	IR + OD	21.57	17.85	55.30	3.57	**39.92**	27.64
AODNet-YOLOXs* [63]	IR + OD	20.66	20.92	54.03	4.76	24.78	25.03
AECRNet-YOLOXs* [13]	IR + OD	23.00	19.96	54.83	2.38	35.81	27.20
RestorNet-YOLOXs* [14]	IR + OD	23.24	18.81	53.71	2.38	35.70	26.77
DS-Net [64]	ODAW	**26.74**	20.54	58.16	7.14	36.11	29.74
IA-YOLO [24]	ODAW	16.20	11.76	41.43	4.76	17.55	18.34
TogetherNet [6]	ODAW	25.70	18.79	57.72	7.14	29.65	27.80
DTRDNet (Ours)	ODAW	26.21	**30.28**	**58.58**	**14.29**	37.37	**33.55**

**Table 3:** Object detection results on the RTTS [31] of real-world hazy images. “*” means the model is trained on the degradation-free images. The best results are bolded, and the sub-optimal results are indicated by underlining.

Method	Type	Class (AP (%))					mAP (%)
		Person	Bicycle	Car	Motorbike	Bus	
YOLOXs [30]	OD	81.78	56.70	70.23	49.48	31.57	57.95
YOLOXs* [30]	OD	80.28	50.75	68.23	41.89	28.89	54.01
DCP-YOLOXs* [12]	IR + OD	81.16	51.34	71.13	47.20	31.09	56.38
AODNet-YOLOXs* [63]	IR + OD	78.16	44.49	65.54	38.88	25.57	50.53
AECRNet-YOLOXs* [13]	IR + OD	80.85	52.75	68.60	46.29	30.43	55.78
RestorNet-YOLOXs* [14]	IR + OD	77.48	51.43	60.92	43.12	29.16	52.42
DS-Net [64]	ODAW	68.81	18.02	46.13	15.15	15.44	32.71
IA-YOLO [24]	ODAW	67.25	35.28	41.14	20.97	13.64	35.66
TogetherNet [6]	ODAW	81.14	54.12	72.39	47.02	32.99	57.53
DTRDNet (Ours)	ODAW	**83.22**	**57.86**	**76.71**	**61.17**	**38.97**	**63.59**

**Table 4:** Object detection results on the VOC-Rain-test of synthetic rainy images. “*” means the model is trained on the degradation-free images. The best results are bolded, and the sub-optimal results are indicated by underlining.

Method	Type	Class (AP (%))					mAP (%)
		Person	Bicycle	Car	Motorbike	Bus	
YOLOXs [30]	OD	80.98	65.88	74.54	70.83	84.35	75.32
YOLOXs* [30]	OD	75.94	63.00	66.92	65.32	73.10	68.86
AirNet-YOLOXs* [51]	IR + OD	80.06	67.81	70.75	69.87	82.77	74.25
RestorNet-YOLOXs* [14]	IR + OD	80.44	68.29	71.68	70.16	82.54	74.62
TogetherNet [6]	ODAW	83.53	69.83	77.88	74.26	**86.41**	78.38
DTRDNet (Ours)	ODAW	**83.87**	**72.52**	**78.59**	**77.66**	86.15	**79.76**

**Table 5:** Object detection results on the VOC-Snow-test of synthetic snowy images. “*” means the model is trained on the degradation-free images. The best results are bolded, and the sub-optimal results are indicated by underlining.

Method	Type	Class (AP (%))					mAP (%)
		Person	Bicycle	Car	Motorbike	Bus	
YOLOXs [30]	OD	81.16	66.64	75.43	70.87	83.28	75.48
YOLOXs* [30]	OD	78.40	64.88	70.80	56.90	81.15	70.43
TKL-YOLOXs* [51]	IR + OD	81.02	70.14	75.53	70.33	84.42	76.29
LMQFormer-YOLOXs* [14]	IR + OD	81.36	70.44	77.57	71.61	84.35	77.07
TogetherNet [6]	ODAW	82.20	69.70	77.72	71.39	85.30	77.26
DTRDNet (Ours)	ODAW	**83.97**	**72.15**	**79.57**	**75.95**	**86.46**	**79.62**

**Table 6:** Object detection results under multiple weather conditions. We show the detection results of clean, hazy, rainy, and snowy images tested on VOC-Clean-test, VOC-FOG-test, VOC-Rain-test, and VOC-Snow-test, respectively. “†” refers to the model trained on VOC-FOG-train, VOC-Rain-train, and VOC-Snow-train. “*” means the model is trained on the degradation-free images. The best results are bolded, and the sub-optimal results are indicated by underlining.

Method	Type	Datasets (mAP (%))				Average
		Clean	FOG	Rain	Snow	
YOLOXs [30]	OD	75.77	74.50	75.50	74.24	75.00
YOLOXs* [30]	OD	79.09	68.86	67.41	70.43	71.45
TogetherNet [6]	ODAW	78.86	76.74	76.78	77.03	77.35
DTRDNet(Ours) †	ODAW	80.16	79.34	78.83	**78.81**	79.28
DTRDNet (Ours)	ODAW	**80.38**	**79.37**	**78.96**	78.80	**79.38**

**Table 7:** The ablation experiments on real hazy dataset RTTS. “Mix” refers to the model trained on the mixed dataset of VOC-FOG-train and VOC-Clean-train. “DD” and “Res” express degradation discriminator and our improved image restoration decoder, respectively. “DD” and “Res” combine to form DDIR. “DCP” stands for degradation category predictor.

Model	Mix	DD	Res	DCP	mAP (%)
TogetherNet [6]	✗	✗	✗	✗	57.53
TogetherNet + Mix	✓	✗	✗	✗	58.87
TogetherNet + Mix + DD	✓	✓	✗	✗	61.98
TogetherNet + Mix + DDIR	✓	✓	✓	✗	62.69
TogetherNet + Mix + DDIR + DCP	✓	✓	✓	✓	63.59

**Table 8:** The ablation experiments on the multiple degradation dataset mixed by VOC-Clean, VOC-FOG, VOC-Rain, and VOC-Snow. “Top1” refers to the precision of degradation classifying. “★” indicates the FLOPs for conducting detection and degradation type classification synchronously.

Model	DD	Res	DCP	mAP (%)	Top1	FLOPs (G)
YOLOXs [30]	✗	✗	✗	74.85	_	13.38
TogetherNet [6]	✗	✗	✗	77.22	_	17.29
TogetherNet + DD	✓	✗	✗	78.77	_	17.29
TogetherNet + DDIR	✓	✓	✗	79.05	_	17.29
TogetherNet + DDIR + DCP	✓	✓	✓	79.24	99.27	17.29/17.56 ^★^

## Data Availability

Data are contained within the article.
